# Clinical Insight into the Precaval Right Renal Artery: A Multidetector Row Computed Tomography Angiographic Study

**DOI:** 10.5402/2013/250950

**Published:** 2013-03-26

**Authors:** Shubha Srivastava, Indra Kumar, C. S. Ramesh Babu, K. K. Gupta, O. P. Gupta

**Affiliations:** ^1^Department of Anatomy, Muzaffarnagar Medical College, Muzaffarnagar 251203, India; ^2^Department of Anatomy, Hind Institute of Medical Sciences, Safedabad, Barabanki 225003, India; ^3^OP Gupta Imaging Center, Bachcha Park, Meerut 250002, India

## Abstract

Variations of course and number of renal vessels are not so uncommon and their knowledge is important for planning of minimally invasive renal surgeries. The earlier literature reports a prevalence of precaval right renal artery between 0.8% and 5%. Normally, the right renal artery passes posterior to the inferior vena cava, but it can also be precaval where it passes anterior to inferior vena cava. The multidetector row contrast enhanced computed tomography angiography allows precise evaluation of renal vasculature. The aim of this retrospective study is to determine the prevalence of precaval right renal artery. Amongst 73 MDCT scans studied, we identified 4 cases of precaval right renal artery with the prevalence being 5.48%, more than what is reported in the earlier literature. We also report a single and dominant precaval right renal artery in one of the cases, which is a rare finding. On the basis of these results, we conclude that precaval right renal artery appears to be more common and so the knowledge of this variant holds a major clinical implication in preventing misinterpretation of radiological images and proper planning of interventional procedures and minimally invasive surgeries.

## 1. Introduction

A sound knowledge of variations of blood vessels is important during operative, diagnostic, and endovascular procedures in the abdomen. Precaval right renal artery (RRA), although rare, is an important variant of renal vascular anatomy and so identifying this anomaly is important for the planning of minimally invasive renal surgery [1]. The knowledge of the renal vascular variations is of extreme importance for the surgeons who approach the kidneys from the retroperitoneal route or laparoscopically for renal transplants [2]. The renal artery variations also show ethnic and racial differences [3]. The occurrence of these variations holds importance because of the gradual increase of interventional radiological procedures, urological vascular, and transplantation surgeries [4]. 

Normally, the right kidney is supplied by the right renal artery passing posterior to inferior vena cava. A Precaval right renal artery (RRA) is defined as a tubular structure with attenuations similar to that of and arising from the aorta or iliac artery that passes anterior to the inferior vena cava (IVC) and terminates in the right kidney [5]. When multiple arteries supply a kidney, the artery with the largest diameter that extends to a given kidney is defined as the dominant renal artery and all other renal arteries are considered as accessory. The present study takes an insight into the prevalence of this anatomical variation and thereby brings awareness to its clinical implications. 

## 2. Materials and Methods

The present retrospective study was done at a single centre in Meerut. No written informed consent was required. The study group consisted of 73 contrast enhanced multidetector row computed tomography (MDCT) angiography scans performed for evaluation of malignancies, abdominal pain, haematuria, or urinary collecting system obstructions. Abdominal aortic dissection, prior urological and renal vascular surgeries, or poorly enhanced scans were not included in the study.

## 3. CT Technique

All 73 patients in the retrospective study underwent MDCT angiographic evaluation (GE optima 660, 64 channels) and received 90–100 mL of nonionic contrast (omnipaque) at the rate of 5 mL/sec intravenously. Scans were obtained from diaphragm to pubic symphysis and 0.625 mm thick sections were obtained. The scans were analyzed in a workstation (AW volume share 4.5). Volume rendered (VR) and maximum intensity projections (MIP) of axial and coronal scans were studied specifically for the presence of precaval RRA. Presence of other renal vascular anomalies, though noted, was not included in the study. 

## 4. Result

In this retrospective study, out of the total 73 randomly selected MDCT scans, only 4 patients had precaval right renal arteries giving a prevalence of 5.48%. Of these 4 patients, 2 were men and 2 were women. All precaval RRAs had origin from the aorta and in no case did the branches of the same renal artery passed both anterior and posterior to IVC. A total of 5 precaval right renal arteries were observed in 4 cases, three of them accessory and two dominant.

**Case 1 .**

**Figure 1:**
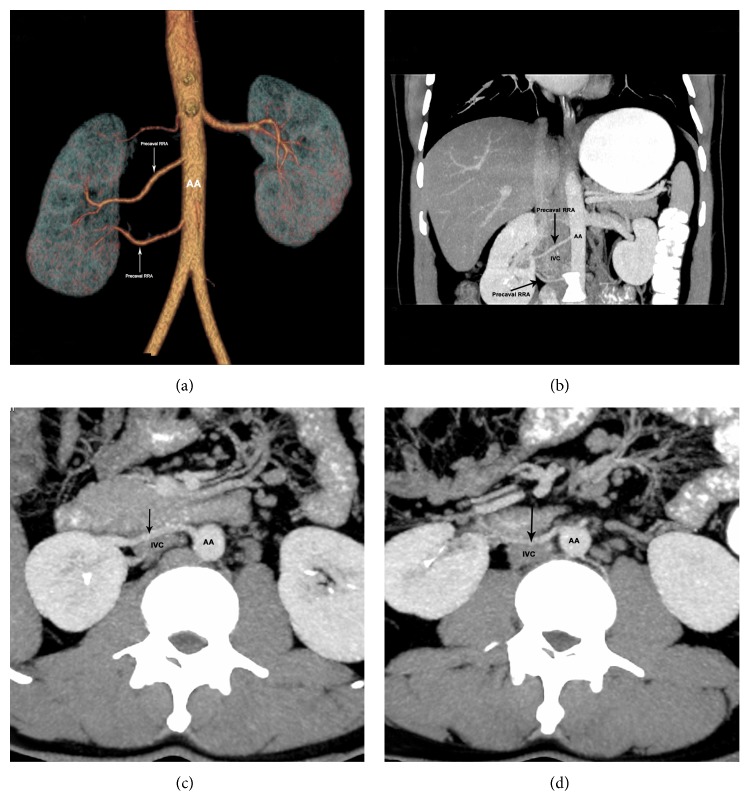
Volume rendered (a) coronal MIP (b) and axial MIP scans ((c)-(d)) showing two precaval RRAs (arrow). The dominant precaval RRA enters the hilum ((a), (b), (c)) and the accessory precaval RRA is going to lower pole ((a), (b), (d)). RRA: right renal artery; AA: abdominal aorta; IVC: inferior vena cava.

A 29-year-old male has 2 precaval right renal arteries with one dominant and one accessory and a superior polar artery, all arising from abdominal aorta. Left kidney is supplied by a single artery (Figures 1(a), 1(b), 1(c), and 1(d)).

**Case 2 .**

**Figure 2:**
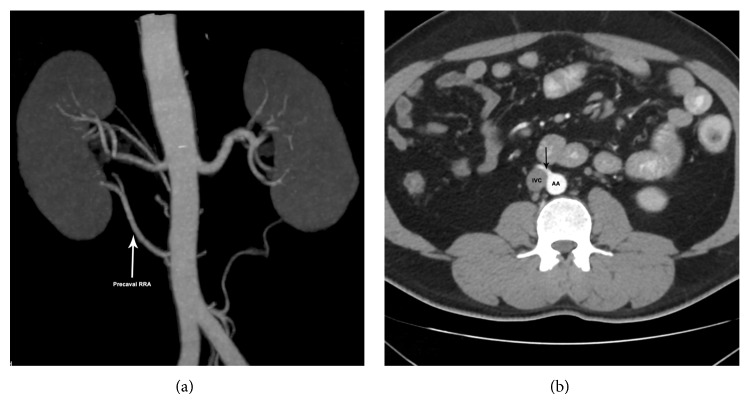
Volume rendered (a) and axial MIP scan (b) showing accessory precaval RRA (arrow) going to lower pole (a). RRA: right renal artery; AA: abdominal aorta; IVC: inferior vena cava.

A 38-year-old male has one accessory precaval right renal artery entering the lower pole and an accessory and a dominant artery entering the hilum. Left kidney is supplied by a single artery (Figures 2(a) and 2(b)).

**Case 3 .**

**Figure 3:**
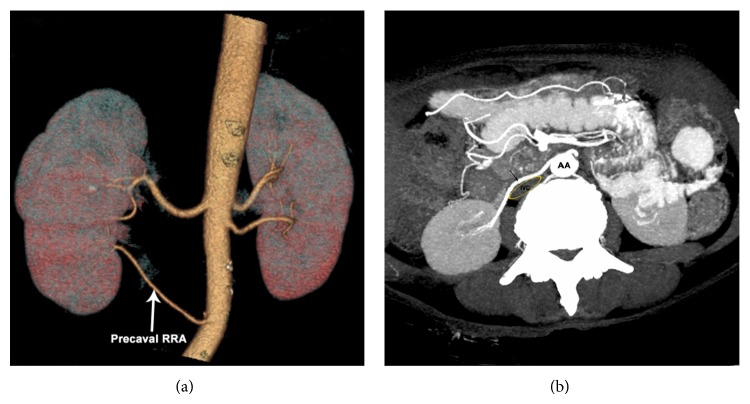
Volume rendered (a) and axial MIP scan (b) showing accessory precaval RRA (arrow). RRA: right renal artery; AA: abdominal aorta; IVC: inferior vena cava (yellow outline).

A 60-year-old female has one accessory precaval right renal artery and a normal dominant right renal artery. Left kidney is supplied by two arteries (Figures 3(a) and 3(b)).

**Case 4 .**

**Figure 4:**
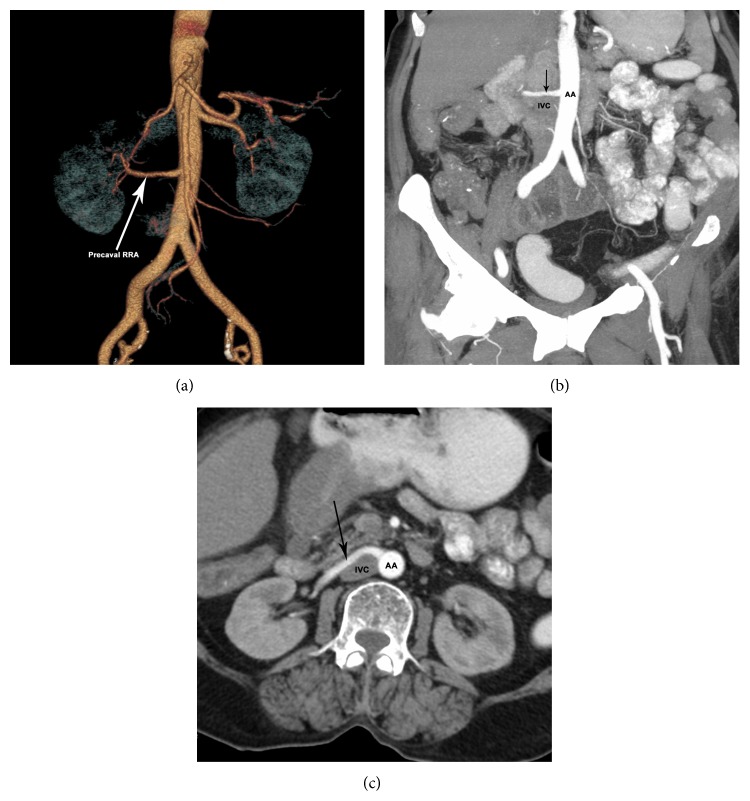
Volume rendered (a) coronal MIP scan (b) and axial MIP scan (c) showing single and dominant precaval RRA (arrow). RRA: right renal artery; AA: abdominal aorta; IVC: inferior vena cava.

A 72-year-old female has a single and dominant precaval right renal artery. Left kidney is supplied by two arteries (Figures 4(a), 4(b), and 4(c)).

## 5. Discussion

During embryonic development, the renal arteries arise from reduction of a series of lateral splanchnic arteries stemming from the aorta supplying blood to the mesonephric kidney. During migration of kidneys from pelvis to lumbar region, kidneys are vascularized by those successive arteries and the final position of the kidney determines the position and number of renal arteries [12, 13]. A precaval RRA is likely to result from a persistent caudal vessel, arising ventrally from the aorta after formation of the inferior vena cava, but before the descent of the gonad [7].

Many studies have shown that volume rendering (VR), maximum intensity projection (MIP), and multiplanar reformation (MPR) can accurately demonstrate accessory renal arteries. Rubin et al. [14] showed that the 3D CT angiography is 100% sensitive in the visualization of accessory renal arteries.

Out of the 73 CT scans reviewed in the present study, 4 showed the presence of precaval RRA with a prevalence of 5.48% which is much higher than that reported by Petit et al. [6], according to whom the prevalence was 0.8% in a series of 380 cases evaluated by ultrasonography (USG) and/or contrast enhanced CT. Yeh et al. [5] with the help of spiral CT described a prevalence of 5% in 186 cases and suggested that most of the precaval RRAs were accessory lower pole arteries. This coincides with our study where we found precaval accessory RRAs in 3 out of 4 cases (Figures 1, 2, and 3). Meng et al. [7] identified three cases of precaval right renal arteries, all accessory lower polar arteries during laparoscopic and endourological procedures, the prevalence being 0.6%. Chai et al. [8] reported one case of precaval RRA in 153 live donors making a prevalence of 0.6%. Bouali et al. [12] retrospectively reviewed 120 arterial phase, contrast enhanced CT scans focusing specifically on the prevalence of precaval right renal artery and reported an incidence of 9.17%. In majority of their cases, lower pole accessory artery was precaval while the main right renal artery was retrocaval. Only in one case they found both main and accessory right renal arteries in precaval position. There was no case of single and main precaval right renal artery or upper pole precaval artery and there are very few cases of these variants in the literature [12]. A study done on 65 Thai renal donors by CT angiography found an incidence of 4.6% [11]. Gupta et al. [10] reported a prevalence of 6% precaval right renal arteries in a study of 50 cadavers which is very close to the incidence observed by us. They also observed the presence of a single and dominant precaval RRA [10]. Single main RRA in a precaval position was observed by Holden et al. [9] in one case out of 100 live donors (1% prevalence) evaluated by MDCT. The findings and the prevalence of precaval RRAs reported in the literature are summarized in Table 1.

An incidental finding of the precaval single main RRA was observed in a contrast enhanced CT of a patient [1], while three RRAs in precaval position were noted intraoperatively [15]. Babu and Gupta [16] also reported a case where the right kidney received two hilar additional renal arteries and both showed a precaval course. Radolinski et al. [17] described a case of a single right renal artery passing anterior to IVC. A cadaveric case report with two precaval RRAs was observed by Raheem et al. [18]. In another case report, Gupta et al. [19] described the occurrence of double precaval right renal arteries in a male cadaver, one main and one accessory, entering the hilum with the accessory artery crossing in front of ureteropelvic junction. Wadhwa and Soni [20] in a cadaveric dissection showed the presence of double precaval right renal arteries entering the hilum. Four precaval right renal arteries of aortic origin with the association of double ureter have also been described in a cadaver [21].

It has been suggested that accessory renal arteries to the lower pole of either kidney that cross the ureteropelvic junction (UPJ) can contribute to obstruction of UPJ. Accessory lower pole right renal artery crossing in front of inferior vena cava and causing obstruction of UPJ has been reported by some authors [5, 22, 23].

The actual incidence of precaval right renal arteries may be higher than that reported in the literature because many such cases might have been missed by an unaware radiologist. It is also possible that the CT technique may have missed other very small accessory renal arteries less than 1-2 mm in diameter [24]. Krishnaveni and Kulkarni [25] reported the presence of five right renal arteries in an ectopic kidney and other vascular anomalies in a single cadaver and all five right renal arteries were in a precaval position, but the authors failed to notice this anomaly and hence did not comment on it. Since renal vascular anomalies are commonly encountered, thorough knowledge of the variations will enhance the proper interpretation of radiological images and performance of safer surgical interventions.

## 6. Conclusion

Knowledge of renal vascular variations has major implication in the clinical practice and it contributes to safety of renal and retroperitoneal surgeries. The present study reported a higher prevalence of precaval right renal arteries (5.48%) than described in the earlier literature. We also report a single and dominant precaval RRA in one case. The occurrence and knowledge of precaval RRAs hold importance as they may be one of the causes for UPJ obstruction and they may be injured during endopyelotomy or may be confused with other vessels such as mesenteric or gonadal at laparoscopy.

## Figures and Tables

**Table 1:** Prevalence and nature of precaval right renal artery (RRA).

Serial number	Name of author	Modality of study	Total number of cases studied	Number of cases and prevalence (%)	Nature of precaval RRAs.
1	Petit et al., 1997 [6]	CECT/USG	380	3 cases (0.8%)	Dominant single precaval RRAs

2	Meng et al., 2002 [7]	Intraoperative/CT	500	3 cases (0.6%)	Lower polar accessory RRAs

3	Yeh et al., 2004 [5]	Spiral CT	186 (retrospective) 3200 (prospective)	9 cases (5.0%) 39 cases (1.2 %)	48 accessory precaval RRAs 4 dominant precaval RRAs

4	Chai et al., 2008 [8]	Live donors Intraoperative	153	1 case (0.6%)	Not available

5	Holden et al., 2005 [9]	Live donors MDCT	100	1 case (1.0%)	Single main RRA

6	Gupta et al., 2011 [10]	Cadaveric dissection	50	3 cases (6.0%)	2 dominant and 2 accessory (one case single dominant precaval RRA)

7	Apisarntharanak et al., 2012 [11]	CT angiography (renal donors)	65	3 cases (4.6%)	1 case single dominant and 2 cases-accessory

8	Bouali et al., 2012 [12]	Spiral CT	120	11 cases (9.17%)	10 accessory lower polar, 1 case both dominant and accessory

9	Present study 2013	MDCT	73	4 cases (5.48%)	2 cases accessory lower polar, 1 case both dominant and accessory, 1 case single dominant
